# The Dual Role of the Antibody Response Against the Flavivirus Non-structural Protein 1 (NS1) in Protection and Immuno-Pathogenesis

**DOI:** 10.3389/fimmu.2019.01651

**Published:** 2019-07-18

**Authors:** Arturo Reyes-Sandoval, Juan E. Ludert

**Affiliations:** ^1^Nuffield Department of Medicine, Jenner Institute, University of Oxford, Oxford, United Kingdom; ^2^Department of Infectomics and Molecular Pathogenesis, Center for Research and Advanced Studies (CINVESTAV), Mexico City, Mexico

**Keywords:** dengue, Zika, flavivirus, NS1 protein, arbovirus, vaccines against flavivirus, immuno-pathogenesis, molecular mimicry

## Abstract

Dengue and Zika viruses are closely related mosquito-borne flaviviruses responsible for major public health problems in tropical and sub-tropical countries. The genomes of both, dengue and zika viruses encodes 10 genes that are translated into three structural proteins (C, prM, and E) and seven non-structural proteins (NS1, NS2A, NS2B, NS3, NS4A, NS4B, and NS5). The non-structural protein 1 (NS1) is a highly conserved glycoprotein of approximately 48–50 KDa. In infected cells, NS1 is found as a homodimer associated with intracellular membranes and replication complexes, serving as a scaffolding protein in virus replication and morphogenesis. NS1 is secreted efficiently from infected cells as a hexamer and is found in patient's sera during the acute phase of the disease. NS1 detection in sera is a valuable diagnostic marker and immunization with NS1 has been shown to protect animal models from lethal challenges with dengue and Zika viruses. Nevertheless, soluble NS1 has been associated with severe dengue and anti-NS1 antibodies have been reported to cross-react with host platelets and endothelial cells and thus presumably contribute to pathogenesis. Due to the implications of NS1 in arbovirus pathogenesis and its relevance as vaccine candidate, we discuss the dual role that anti-NS1 antibodies may play in protection and disease and the challenges that need to be overcome to develop safe and effective NS1-based vaccines against dengue and Zika.

## Introduction

Dengue is the most important mosquito-borne viral disease in humans. It is endemic in over 100 countries and it is estimated that nearly 2/3 of the world's population lives in risk areas for this disease. The dengue virus (DENV) is classified as part of the genus *Flavivirus* within the family *Flaviviridae* and is transmitted to humans mainly by two mosquito species, *Aedes aegypti* and *Aedes albopictus* (1). Other mosquito-borne flaviviruses causing disease in humans are the Yellow fever virus, the West Nile virus, the Japanese encephalitis virus and the Zika virus.

There are four DENV serotypes circulating around the world and all can cause disease. Whilst most DENV infections are asymptomatic, they can present clinical signs such as high-degree fever, headache, muscle, joint pain, and rash; clinical signs are usually fully resolved within 5–7 days in dengue fever (DF). However, DF can evolve in a fraction of the patients to a life-threatening form of the disease, severe dengue (SD), characterized by bleeding, plasma leakage and organ impairment (2). DENV infection confers life-long protection against the homologous serotype. However, a secondary infection with a heterologous serotype is a risk factor for the development of severe dengue (3, 4). The antibody-dependent enhancement (ADE) has been pointed as a major mechanism underlying the increased risk of severe dengue during secondary infections (4). Despite the great burden associated with dengue, so far there is no specific treatment for this disease (5) and, unfortunately, the current licensed tetravalent live-attenuated vaccine has been associated with predisposition to severe dengue when applied to DENV-naive people (6).

The DENV virion is enveloped with ~50 nm in diameter and the genome consists of a single-stranded RNA molecule of positive polarity of approximately 11 Kb (7). The DENV genome encodes for 3 structural proteins (capsid, C; precursor membrane and membrane prM/M; envelope, E) and for 7 non-structural proteins (NS1, NS2A, NS2B, NS3, NS4A, NS4B, NS5), all derived from a polyprotein of around 3,400 amino acids by proteolytic processing (7). Viral replication occurs in the cytoplasm in association with the rough endoplasmic reticulum (RER) and involves both viral NS proteins and cellular proteins, for replication, translation, and encapsidation of the genome (7). Finally, in vertebrate cells, mature virions are secreted to the extracellular media, along with the NS1 protein, following a classical secretory route that involves the Golgi complex and the previous cleavage of the prM protein by the host protease furin (8).

## The Multiple Properties of the Flavivirus NS1 Protein

The DENV NS1 is a glycoprotein of around 46–50 KDa, that shows high conservation among the 4 DENV serotypes and even among various other arthropod-borne flaviviruses (9–11). Mature monomeric NS1 is released into the lumen of the ER after cleavage from E and NS2A. In the infected cell, NS1 is found mainly associated to intracellular membranes and organelles induced by the virus infection. However, a fraction of NS1 can also be found associated with lipid rafts on the plasma membrane or soluble, secreted into the supernatant (12, 13). Membrane-associated NS1 is dimeric, with well-defined hydrophobic and hydrophilic faces, facing the ER membrane and the ER lumen, respectively (14). Secreted NS1 is an open barrel hexamer associated with lipids (14, 15). Three distinct domains have been identified along the structure of NS1; a β-roll domain comprising the first 29 amino-terminal residues, followed by a “wing” domain, comprising positions 30–180 and finally a β-ladder domain, constituted by the carboxy-terminal residues 181–352. While the wing and β-ladder domain are basically hydrophilic in nature, the β-roll domain is hydrophobic and likely to interact with cell membranes (13). NS1 has been found as an organizational protein of the viral replication complexes essential for viral viability, even though its exact role in DENV the replication is not yet fully understood. It has been suggested that intracellular NS1 is a necessary cofactor for viral RNA replication and virion morphogenesis and may also play a role in the modulation of the innate immune response (13, 14).

NS1 is found at high levels (in the order of μ/ml) in the sera of infected patients early during infection and extensive evidence suggests that NS1 is related to SD pathogenesis (9–11). Fundamental characteristics that distinguish SD from the more benign forms of the disease are hemorrhage, coagulopathy, and a sharp increase in vascular permeability (2). Disease severity have been found to correlate with high levels of circulating NS1 in patient's sera (16–18). To explain the direct participation of NS1 in SD, several mechanisms, such as the capacity of NS1 to form complexes with prothrombin/thrombin, have been proposed (17). More recent evidence indicates that DENV NS1 has the capacity to directly induce glycocalyx degradation as well as destabilization of tight junctions in a tissue-specific manner, suggesting a direct participation of NS1 in the endothelial plasma leakage observed in patients with SD (19, 20). Soluble NS1 has also been shown to activate key cell components of vascular homeostasis such as macrophages, mononuclear peripheral cells and platelets via direct interaction with Toll-Like Receptor 4 (TLR4) (21, 22). The NS1-mediated activation of macrophages and mononuclear cells leads to secretion of pro-inflammatory cytokines, known to alter tight junctions (23), while the activation of platelets leads to increased platelet aggregation, adhesion to endothelial cells and phagocytosis by macrophages. In addition, pre-incubation of hepatocytes with soluble NS1 enhances the replicative capacity of these cells for dengue, suggesting a role for NS1 in the modulation of innate immune responses (13). All these results strongly support the notion that soluble NS1 directly participates in the plasma leakage, thrombocytopenia, and hemorrhages associated with SD.

## ANTI-NS1 Antibodies Cross Reactivity With Host Molecules

Soluble NS1 elicits strong humoral responses in the host. Anti-NS1 antibodies can either be protective or deleterious to the host, as NS1 antibodies can recognize host factors by molecular mimicry to cause tissue damage and impair physiological functions. The notion that anti-NS1 antibodies do participate in dengue pathogenesis resulted from experiments by Falconar, who showed that anti-NS1 monoclonal antibodies cross-react with human fibrinogen, thrombocytes and endothelial cells and produce hemorrhage in a mouse model (24).

**Figure 1:**
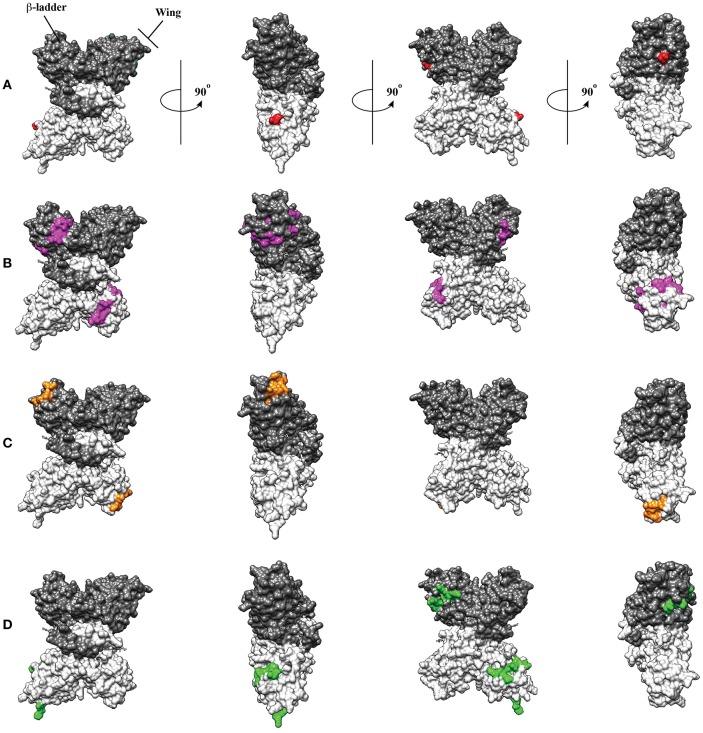
Molecular model of dengue virus NS1 showing regions involved in molecular mimicry with LYRIC in endothelial cells **(A)**, with the protein disulfide isomerase in platelets **(B)** and plasminogen **(C)**. Those two last epitopes overlap in one position (311). An immunodominant epitope presumably involved in protection (28) is also shown **(D)**. Interestingly, this last epitope does not overlap with any of the epitopes related to molecular mimicry. Monomers are shown in gray and white and wing and ladders domain are indicated.

Plasma leakage is one of the key clinical manifestations of severe dengue and a major target for anti-NS1 antibodies are endothelial cells, key players in maintaining vascular homeostasis. In a pioneering article on the subject, Lin et al. (25) reported that sera collected from dengue patients contained antibodies able to induce damage to HUVEC endothelial cells via caspase activation and induction of apoptosis. Pre-treatment with recombinant NS1 reduced the damaging capacity of the sera. The same group later reported that binding of anti-NS1 antibodies to HMEC-1 endothelial cells causes the expression of proinflammatory cytokines such as IL-6, IL-8, and MCP-1 and cell activation as indicated by the expression of adhesion molecules such as ICAM-1 (26). The molecular bases of the cross reactivity of anti-NS1 antibodies with endothelial cells have been partially defined. Using HUVEC cells, Liu et al. (27) found that the human protein LYRIC (lysine rich CEACAM1 co-isolated) is a target recognized by anti-NS1 cross-reactive antibodies in endothelial cells. The binding of cross-reactive anti-NS1 monoclonal antibodies (mAbs) to LYRIC enhanced apoptosis and complement-dependent cell cytotoxicity, indicating that the recognition of LYRIC is indeed associated to endothelial cell damage. Finally, an epitope located in a disordered loop of the wing domain of NS1 (116–119) has been deemed responsible for the cross reactivity between NS1 and LYRIC (Figure 1A). Of note, LYRIC plays a role in the activation of various signaling pathways, including the NF-κB; in agreement, the reduction of apoptosis and nitric oxide (NO) production of endothelial cells mediated by anti-NS1 antibody binding can be significantly reduced if cells are treated with inhibitors of the NF-κB pathway (29, 30).

Anti-NS1 cross reactive antibodies may also play a role in the liver damage observed in patients with dengue, as suggested by observations using a murine model. Inoculation of mice with recombinant DENV NS1, but not with JEV NS1, resulted in the production of antibodies capable of recognizing naïve mouse liver endothelial cells and interestingly, not kidney endothelial cells. Moreover, mice inoculated with rNS1 or passively immunized with anti-NS1 IgG, showed altered transaminase (AST and ATL) levels, but not indication of kidney damage (31); yet, there is not obvious explanation for the organ specific damage cause by anti-NS1 antibodies.

Coagulopathy and thrombocytopenia are also features associated with SD. Anti-NS1 antibodies have also been reported to target platelets, thrombin and plasminogen. The protein disulfide isomerase (PDI) on platelets is recognized by antibodies that recognize a peptide of DENV NS1 corresponding to amino acid residues 311–330, located in the C terminal region, in the β-ladder domain (Figure 1B). These antibodies are present in the sera of naturally infected patients and upon binding to platelets are capable of inhibiting isomerase activity and promote platelet aggregation (32, 33). In addition, platelets opsonized by anti-NS1 antibodies are more readily phagocytosed by macrophages (34). Finally, anti-NS1 antibodies also recognize components of the coagulation pathways, pointing to a role of these antibodies in the hemorrhagic disorders associated with dengue (35). After immunization of mice with DENV rNS1, several mAbs capable of recognizing human plasminogen could be isolated (35). These mAbs not only bind plasminogen but also induce its activation and conversion to plasmin due to catalytic properties. Again, the cross-reactive epitope on NS1 raising the higher affinity mAbs (305–311) is in the β-ladder, C-terminal region of the protein (Figure 1C).

## The NS1 Protein As a Vaccine Candidate

Traditionally, dengue vaccines have been developed using the envelope (E) and pre-membrane proteins (prM) of dengue virus (DENV) as immunogens. This approach has resulted in the first licensed vaccine with regulatory approval in various countries (36). However, alternative strategies are still pursued due to the risk of vaccine-related ADE induction made evident during follow up studies of the CYD-TDV leading vaccine candidate (37). NS1 has been an attractive candidate for many years due to (a) lack of presence on the virion's surface, resulting in lower risk of inducing antibodies with ADE potential; (b) high degree of conservation amongst DENV serotypes; (c) high levels of NS1 protein secretion of up to 50 μg/ml of plasma, which correlates with severity during DHF/DSS (13, 38) and acts as a viral toxin, thus becoming a potential vaccine target; (d) early evidence that recovered patients have high titers of anti-NS1 antibodies; (e) high levels of immunogenicity and evidence of protection against DENV infection in mice upon NS1 vaccination and the involvement of antibodies and CD4^+^ T cells (39). This is in spite of the controversial role that anti-NS1 antibodies can play, such as the cross reactivity with endothelial cell surface proteins leading to apoptosis (25–27, 29, 30), platelet cross reactivity causing dysfunction and tendency to bleed (32, 33) and reactivity with proteins of the coagulation cascade, such as thrombin (40) (Figures 1A-D). Initial observations made by Schlesinger et al. during mid 1980s of the protective efficacy of the glycoprotein gp48 (NS1) against Yellow Fever in mice (41) and macaques (42) led to the seminal work showing that immunization with DENV-2 NS1 protein was able to elicit protection against a homologous DENV infection (43). Recombinant viral vectors in the form of vaccinia virus made an early entry in the NS1 vaccine field, and vaccine efficacy was demonstrated using mouse encephalitis DENV models (44). By 2003, DNA vaccines had become a trend in vaccinology and attempts to induce immunity against DENV without risk of ADE prompted the development of DNA vaccines expressing NS1. Co-administration of DNA-NS1 with IL-12 as genetic adjuvant demonstrated efficacy against a DENV-2 challenge (45). Importantly, it became evident for genetic vaccination that leading sequences are of major importance in targeting the protein to the secretory pathway to enhance antibody responses and improve efficacy of DNA-NS1 vaccines (46, 47), an early lesson for future flavivirus vaccine design using DNA or viral vectors (48). NS1 has eventually been produced in bacteria and used as vaccine in presence of adjuvants. Inclusion of *E. coli* ETEC heat-labile toxin (LT_G33D_) as adjuvant has been shown to yield better efficacy against a DENV-2 challenge than traditional adjuvants like Alum or Freund's adjuvant (49). Vaccinations with NS1, as well as NS1-immune sera or mAbs can protect against a lethal DENV challenge, thus underscoring the potential of NS1-based vaccines. NS1 vaccination may result in cross-reactivity with host proteins of vaccinees, hence making this a challenging approach. However, mAbs have been useful to identify non cross-reactive sequences through epitope mapping using phage display that are yet able to show protective efficacy against a challenge with DENV (50). The latter research highlights the potential of monoclonal antibodies and structural-guided vaccinology to design NS1 vaccines with ability to protect against infection with yet a reduced potential of adverse reactions due to cross reactivity with self-antigens. NS1 has also proven valuable in immunity and vaccine development against Zika virus (ZIKV) in the context of potential increased severity through ADE between the two highly similar ZIKV and DENV. Human monoclonal antibodies against ZIKV NS1 have been isolated and shown protective efficacy in mouse models through the engagement of FcγR without the requirement of virus neutralization (51). These results lead to a subsequent demonstration of protective efficacy of an NS1-based DNA vaccine in a lethal ZIKV challenge model (52).

## Concluding Remarks

The mechanisms underlying severe dengue are not fully understood, but certainly involve a combination of viral virulence, host genetics, and immunopathology. For as long as ADE has been recognized as an immune mechanism that promotes severe dengue (DHF/DSS), efforts have been made to find alternative DENV vaccine strategies to induce immune responses against antigens other than structural components of the DENV virion. The observation that both, structural (prM and Envelope) proteins and the non-structural protein 1 (NS1) elicit strong humoral immune responses in infected individuals has prompted the development of vaccines against both DENV components. Recently, it was shown that IgG anti-NS1 antibodies in sera from a phase II clinical trial were effective in preventing endothelial damage cause by NS1 in an *in vitro* model (53). These antibodies will not only prevent ADE but can confer NS1 cross protection with the other 3 DENV serotypes. Nevertheless, development of NS1 vaccines is yet a challenging approach due to the cross-reactive immune responses between NS1 and self-antigens in endothelia, platelets and clotting factors. NS1 structural studies and availability of monoclonal antibodies are permitting the identification of peptide sequences within NS1 domains that are suitable to generate immunity against DENV with a decrease in cross reactivity to self-antigens. Interestingly, anti-JEV NS1 antibodies, used as controls in various experiments with anti-NS1 antibodies (26, 31) do not cause any cell damage. Structural studies showed differences between the JEV NS1 and the DENV NS1 in the C-terminal, β-ladder domain, where cross-reactive epitopes to platelets and plasminogen are located (54). Indeed, antibodies to chimeric JEV-DENV NS1 protein showed reduced cross-reactivity while still conferring protection in a mouse model (55). Finally, despite the compelling evidence obtained *in vitro* and with animal models, indicating a role for anti-NS1 antibodies in SD pathogenesis, it is wise to keep in mind that plasma leakage, thrombocytopenia, and other vascular clinical signs are all transient and last only for a few days, much shorter than the presumed half-life of any antibody. Thus, additional research is still needed to fully understand the association between anti-NS1 antibodies and dengue pathogenesis in patients and the extent to which molecular mimicry need to be avoided in NS1 based vaccines.

## Author Contributions

All authors listed have made a substantial, direct and intellectual contribution to the work, and approved it for publication.

### Conflict of Interest Statement

The authors declare that the research was conducted in the absence of any commercial or financial relationships that could be construed as a potential conflict of interest.
